# Notes on Health Resorts

**Published:** 1901-01-19

**Authors:** 


### THE INSTITUTIONAL WORKSHOP.

### NOTES ON HEALTH RESORTS.

### STRATHPEFFER.

ON the eastern coast of the northern part of Scotland lies a great indentation which goes by the name of the Moray Firth, and running in still further from the town of Cromarty westwards, deep into the heart of the highlands, is a narrow waterway, Cromarty Firth, at the head of which stands Dingwall, the county town of Ross-shire. Opening on to this arm of the sea at Dingwell is a valley which runs up westwards under the protecting wing of the great Ben Wyvis, a mountain 3,429 feet high, by which it is sheltered from the north, and near the head of this, in sharp contrast with all its surroundings, lies the fashionable watering place of Strathpeffer. Far away as it seems, however, and admirably as it is situated for the purposes of the health seeker who wishes to go back to nature to restore his jaded faculties, it is reached easily enough by rail, lying as it does only about twenty miles north-west of Inverness. In many ways Strathpeffer offers advantages to those who look for change and pure air. It is well protected from the north by Ben Wyvis and other hills, on the east it is not far from the sea, while to the west it is on the very verge of a large open country of rocks and streams, of moors and deer forests. Moreover, it is within the belt of country which is under the climatic influence of the Gulf Stream, so that the cold in winter is by no means as severe as its latitude would suggest. It is indeed strongly recommended by the medical men practising there as a winter resort, the special advantages claimed for it arising from the clearness of the air, the absence of fog, and the amount of bright sunshine which prevails. The special attraction, however, which Strathpeffer offers, and that which chiefly helps to give to it its high place among health resorts, lies in its waters, especially those which are charged with sulphur. The Strathpeffer waters are of two kinds, sulphur and chalybeate, both of which are different from the waters met with at most other spas—the sulphur water principally in the absence of those saline constituents which form so prominent a feature in the product of the great English sulphur springs at Harrogate, and the chalybeate water in the fact that the iron occurs in the form of a simple carbonate kept in solution by the free carbonic acid which the water contains. A special advantage which is claimed for the Strathpeffer sulphur water is that not only is it very highly charged with sulphur, but that this element exists in it in the form of sulphuretted hydrogen in a far larger degree than at other spas. Thus, in Dr. Fox's little book on Strathpeffer Spa the following table is given, comprising not merely the total amount of sulphur, but the amount in its different forms at four sources: at Challes, which is said to be the strongest sulphur spring in Europe, at the Harrogate Old Sulphur Well, and at the two springs at Strathpeffer :—

Grains of Sulphur per Gallon.

| | As Sulphide or Sulphydrate of Sodium. | As Sulphuretted Hydrogen. | Total. |
|---|---|---|---|
| Eau de Challes ... ... (Garrigon) | 12·8 | 1·0 | 13·8 |
| Strathpeffer—Cromartie Well (Maxwell) | 0·95 | 6·51 | 7·46 (maximum 11·1) |
| Morrison Well ... ... (Maxwell) | 0·25 | 6·23 | 6·48 |
| Harrogate Old Sulphur (Thorpe) | 2·90 | 3·56 | 6·46 |

From this it would appear that not only is the Strathpeffer water stronger in sulphur than Harrogate water, but that, although beaten in total sulphur by Challes, it is far stronger even than that spring in the proportion of the sulphur which exists in the form of sulphuretted hydrogen. How far this is an advantage may, perhaps, be open to question, and probably some will doubt whether the claims made as to the superior efficacy of the sulphuretted hydrogen water over those in which the sulphur occurs in the form of sulphide can be quite substantiated. But there is certainly one point in which the Strathpeffer water is almost unique, and that is that, while highly charged with sulphur, it is free from that large admixture of chlorides which make one class the springs of Harrogate almost as much with the muriated as with the sulphur waters. There can be no doubt of the great advantage of having such a pure sulphur water. Nothing can deprive a strongly muriated water of its excess of salt or make it suitable for those with whom this disagrees; but it is easy enough to add any required salines to the waters of Strathpeffer, and thus obtain a considerable variety of therapeutic effects.

The waters of Strathpeffer are employed in the treatment of chronic gouty conditions, various forms of chronic rheumatism so called, a large range of dyspeptic troubles connected with catarrhal conditions and disorders of the liver, and various chronic skin diseases. The possession of a carbonated chalybeate water which may be used as a tonic is found to be of great advantage.

The sulphur water is used both internally and externally, but it is on its internal use that the greatest reliance is placed. As at most watering places which are intelligently managed, success and popularity have led to the development of various special facilities for the treatment of the class of patients frequenting the place by methods not directly connected with its waters. Of late years much has been done to provide douches of various kinds: moor or heat baths, Russian, needle and pine baths, massage and manipulations of various kinds have been employed, and lately the Nauheim treatment for cases of cardiac disease has been made a feature of.

During the summer season, which lasts from April to October, there is a band; there are plenty of visitors in the place itself, and excursions can be made on every side, so that it is an attractive place enough even for those who are not undergoing the "cure."

